# Identification of a rhodopsin gene mutation in a large family with autosomal dominant retinitis pigmentosa

**DOI:** 10.1038/srep19759

**Published:** 2016-01-22

**Authors:** Xinping Yu, Wei Shi, Lulu Cheng, Yanfang Wang, Ding Chen, Xuting Hu, Jinling Xu, Limin Xu, Yaming Wu, Jia Qu, Feng Gu

**Affiliations:** 1 School of Ophthalmology and Optometry, Eye Hospital, Wenzhou Medical University, State Key Laboratory Cultivation Base and Key Laboratory of Vision Science, Ministry of Health and Zhejiang Provincial Key Laboratory of Ophthalmology and Optometry, Wenzhou, Zhejiang 325027 China; 2 Department of Ophthalmology, Beijing Children’s Hospital, Capital Medical University, Beijing 100045 China; 3 Zhejiang Key Laboratory of Medical Genetics, School of Laboratory Medicine and Life Sciences, Wenzhou Medical University, Wenzhou, Zhejiang 325035 China; 4 Department of Ophthalmology, The First Affiliated Hospital of Zhengzhou, Zhengzhou University, Henan 450052 China; 5 Department of Ophthalmology, the First Affiliated Hospital of Wenzhou Medical University, Wenzhou, Zhejiang 325000 China

## Abstract

Retinitis pigmentosa (RP) is a genetically highly heterogeneous retinal disease and one of the leading causes of blindness in the world. Next-generation sequencing technology has enormous potential for determining the genetic etiology of RP. We sought to identify the underlying genetic defect in a 35-year-old male from an autosomal-dominant RP family with 14 affected individuals. By capturing next-generation sequencing (CNGS) of 144 genes associated with retinal diseases, we identified eight novel DNA variants; however, none of them cosegregated for all the members of the family. Further analysis of the CNGS data led to identification of a recurrent missense mutation (c.403C > T, p.R135W) in the rhodopsin (*RHO*) gene, which cosegregated with all affected individuals in the family and was not observed in any of the unaffected family members. The p.R135W mutation has a reference single nucleotide polymorphism (SNP) ID (rs104893775), and it appears to be responsible for the disease in this large family. This study highlights the importance of examining NGS data with reference SNP IDs. Thus, our study is important for data analysis of NGS-based clinical genetic diagnoses.

Retinitis pigmentosa (RP) is the most common form of inherited retinopathy, with a prevalence of approximately 1 in 3500^1^. It is a clinically and genetically heterogeneous group of eye diseases and one of the leading causes of blindness in the world. Clinically, the age at onset of symptoms is highly variable and ranges from childhood to mid-adulthood. Genetically, RP displays all three modes of Mendelian inheritance—autosomal dominant (adRP), autosomal recessive (arRP), and X-linked (XLRP)—, as well as digenic and mitochondrial inheritance modes^2^. Thus far, at least 280 genes have been identified as the cause of one form or another of inherited retinal disease^2^,^3^,^4^,^5^. This complex of genes and clinical features complicates the corresponding clinical diagnoses. For example, although rhodopsin mutations usually cause dominant RP, other rare rhodopsin mutations cause arRP^6^. Mutations in *ABCA4* are found in most patients with autosomal-recessive Stargardt disease, and studies have shown that mutations in the *ABCA4* gene are responsible for a wide variety of other retinal dystrophy phenotypes, such as cone-rod dystrophy (CRD) and RP^7^,^8^.

Approaches based on stem cell and gene therapy hold a great deal of promise for treatment of retinal disease; however, currently, no effective therapies have been identified^3^,^9^,^10^,^11^. Thus, identifying new genes and mutations for clinical genetic diagnosis and prenatal diagnosis of RP is crucial. Because at least 280 genes have been identified as the cause of various forms of inherited retinal disease, identifying underlying mutations with traditional methods has been challenging^3^,^12^. Next-generation sequencing (NGS) is a highly versatile and effective approach to detecting novel disease-causing genes and mutations in families with different diseases, including retinopathies^13^,^14^. Our previous study provided support for using NGS as an effective approach to distinguishing cone-rod dystrophy and Stargardt disease, two inherited retinopathies with overlapping clinical symptoms^7^.

In the present study, patients and unaffected individuals from a five-generation family with fourteen individuals diagnosed with RP were recruited. We sought to identify the underlying genetic defect in this family by capturing next-generation sequencing (CNGS) and Sanger sequencing. These approaches led to identification of eight novel DNA variants, but the variants did not cosegregate with all affected individuals in the family, indicating none of them was the disease-causing mutation. Thus, re-analysis of the data was necessary because the disease-causing gene may already be identified or be outside of the panel of the captured genes. To rule out the first possibility, we retrieved the data from the CNGS. We observed a recurrent mutation (p.R135W) in the rhodopsin (*RHO*) gene with reference SNP ID (rs104893775, http://www.ncbi.nlm.nih.gov/projects/SNP/snp_ref.cgi?rs=104893775).

## Results

### Clinical data

**Figure 1:**
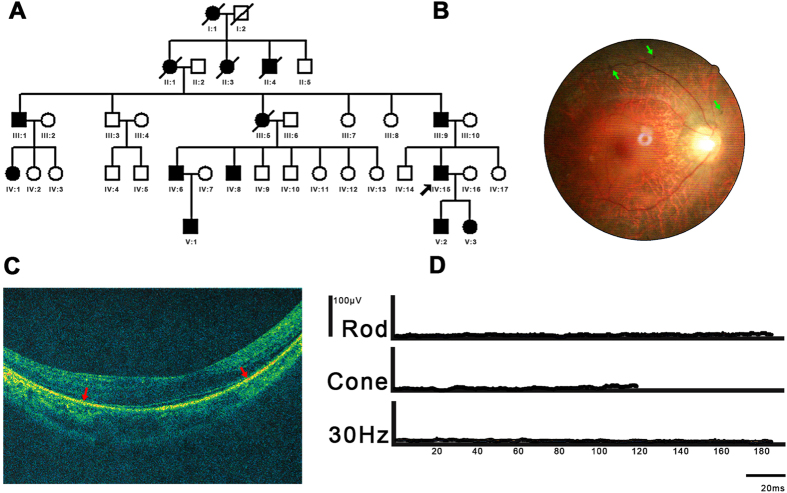
Pedigree and clinical manifestations. (**A**) Pedigree of autosomal-dominant RP family. Black circles (females) and black squares (males) represent affected individuals. Unaffected individuals are not shaded. Black lines denote deceased. An individual is identified by generation number and the numbers below the symbols. The arrow marks the proband. (**B**) Color fundus photographs demonstrate slight intraretinal bone spicule pigmentation has been observed. (**C**) Outer retina layers of the patient on OCT are disrupted and disorganized, indicating the dysfunction of the photoreceptors. (**D**) Non-recordable ERG has been detected, indicating the functions of both cone and rod are severely reduced.

The family in this RP study included 14 affected individuals in a five-generation family (Fig. 1A) originating from Henan province, China. The proband was a 35-year-old male (indicated with black arrow in Fig. 1A). His decimal best-corrected visual acuity (BCVA) was 0.3 (Table 1). Fundus examination revealed slight intraretinal bone spicule pigmentation (green arrow in Fig. 1B). 3-D optical coherence tomography (OCT) scans revealed the degree of thinning of the outer retinal layer (red arrow in Fig. 1C), indicating the dysfunction of the photoreceptors. Electro-retinogram (ERG) results showed both cone and rod functions were seriously affected (Fig. 1D). Night-blindness was also reported. The clinical data of the proband and family members are shown in Fig. 1, Figure S1, and Table 1. According to the available clinical data, three affected individuals had RP-specific symptoms, including night blindness, thinning of the outer retinal layer and serious effects of both cone and rod functions. As for the early stages of the disease, recordable cone function may still be observed (Figure S1). All three affected individuals had similar levels of best corrected visual acuities while the corresponding astigmatism was dramatically different. Thus, based on the clinical manifestations, the diagnosis of RP is appropriate.

### DNA variants in CNGS

We used a CNGS platform for screening 144 genes associated with retinal diseases. After initial analysis (http://122.228.158.106/exomeassistant) of candidate mutations, we identified eight distinct DNA variants in the samples (Table S1). We then performed cosegregation analysis to determine whether these DNA variants could be responsible for the disease. Sanger sequencing of the amplified fragments harboring the candidate mutations was performed to test for cosegregation. None of the eight mutations cosegregated with all affected individuals in the family.

### *RHO* mutation screening

Because no cosegregating mutation was identified in the initial sequencing data analysis, we re-analyzed the CNGS data from the CNGS experiment. Because it has been reported that mutations in the *RHO* gene account for 7.7% to 40% of all adRP cases in different populations^6^,^15^,^16^, we investigated whether the mutation in the family of study was located in the area of missing sequence coverage in the *RHO* gene. This strategy was similar to one used in our recent study in which we successfully filled in missing sequence data for the *OPA1* gene from CNGS data while searching for disease-causing genes linked with autosomal dominant optic atrophy^17^. In this present study, the CNGS data showed good coverage of the *RHO* gene (Table S2). A single *RHO* DNA variant was detected in the sequence (c. 403C >T, p.R135W), with a reference SNP ID (rs104893775).

**Figure 2:**
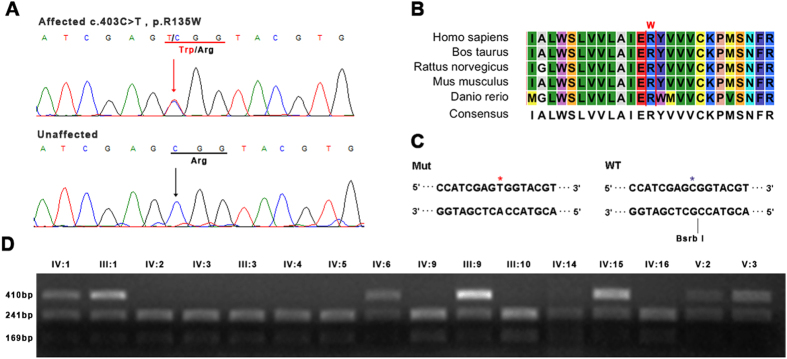
Mutation analysis. (**A**) DNA-sequence chromatograms of the affected family members with RP disease. The heterozygous peak of the mutation is indicated by red arrows. (**B**) Multiple-sequence alignment in RHO from different species indicating the mutation (p.W135R) in RP patient is located within a highly conserved region. (**C**) Schematic diagram of RFLP for the affected and unaffected individuals. (**D**) RFLP results showed p.W135R mutation in *RHO* cosegregated with all affected individuals in the family and was not observed in any of the unaffected family members. The purified PCR products were digested with Bsrb 1, showing the unaffected individual with 240 and 169 bp fragments as opposed to the affected individual with 241, 169 and 410 bp.

Surprisingly, it has been reported that the p.R135W mutation in *RHO* is a disease-causing RP mutation in different populations^18^,^19^,^20^,^21^,^22^. Thus, we used Sanger sequencing to confirm that the proband had the mutation (Fig. 2A). We found that codon 135, where the mutation (p.R135W) occurred, was located within a phylogenetically conserved region (Fig. 2B). Then, we performed a cosegregation study using the restriction fragment length polymorphism (RFLP) method. Specifically, fragment harboring the mutation was amplified with genomic DNA from the family members. The purified PCR products were digested with *Bsrb* 1, which causes differential patterns of bands on agarose gels between the unaffected and affected individuals. Compared to the unaffected individuals with fragments of 241 and 169 bp, affected individuals had 241,169 and 410 bp fragments (Fig. 2C). RFLP analysis confirmed this mutation cosegregated with all affected individuals in the family and was not present in any of the unaffected family members (Fig. 2D) or the 1402 normal controls (from in-house exome databases with 1402 samples from a Chinese population).

In addition, we screened the remainder of the coding sequence of *RHO* (Table S3), and no additional sequence changes were detected. Furthermore, we used online bioinformatics software tools designed to distinguish between functionally neutral and deleterious amino acid changes in mutagenesis studies and human polymorphisms. We predicted the amino acid substitution in *RHO* could have a phenotypic effect and found that the substitution at position 135 from R to W to be deleterious (Table S4). Thus, the p.R135W mutation in *RHO* is a disease-causing mutation in this family.

## Discussion

NGS offers a cost-effective approach to detecting disease-causing genes for clinically and genetically heterogeneous disorders or drug resistant genes^23^,^24^. However, analytical data analysis for identifying mutation from NGS data remains a challenge because NGS could provide massive amounts of data^25^. In addition, the success of using NGS data may be inconsistent because of different disease inheritance modes. Specifically, because the pathogenic mutations for autosomal-recessive disorders may be recorded in the dbSNP database, DNA variants with SNP IDs could be assigned as candidate mutations^26^,^27^. As for autosomal-dominant disorders, traditionally, to identify a mutation, novel DNA variants from NGS data are prioritized for selection as disease-causing genes because only a single mutation would be responsible for the disease. In this study, we showed ignoring DNA variants with reference SNP IDs may lead to missing the disease-causing mutation. The incorrect conclusion—that no mutation is present—may lead to the further time and expense of whole-exome or whole-genome sequencing. This study highlights the importance of suitable treatment of NGS data with reference SNP IDs for clinical genetic diagnosis.

For clinical laboratories, determining which sequence variants are pathogenic is difficult, particularly if no simple functional assays are readily available to determine the phenotypic effects of specific variations^28^. The *RHO* DNA mutation (c. 403C >T, p.R135W) has been reported in at least five studies with different populations^18^,^19^,^20^,^21^,^22^. In this present case, it cosegregates with all affected individuals in the large family examined and was not observed in any of the unaffected family members or normal controls. The functional studies also showed that Arg135 residue is an important interaction site with transducin^29^. The bovine p.R135W rhodopsin mutant was unable to activate the G protein *in vitro*, contrary to the wild-type rhodopsin^30^. In addition, the glycosylation state of the p.R135W rhodopsin mutant is the most defective^19^. As for the two different mutations at Arg135 residues—R135L and R135W—, both result in diffuse severe RP disease, but R135W causes more severe and a more rapidly progressive RP than does R135L. Therefore, p.R135W could be considered a causative mutation beyond reasonable doubt, despite having a reference SNP ID (rs104893775). For newly discovered or rare gene variants with a reference SNP ID, we still need to examine the candidate gene carefully. As more data are deposited and more papers published, the assignment of the mutation will become easier.

In our previous study, we showed missing sequence coverage of some exons in the *PROM1* gene from CNGS-based molecular diagnosis of putative Stargardt disease, one of the most common genetic forms of juvenile or early adult onset macular degeneration^7^. Further, with deep sequencing, missing coverage of some regions is common^31^. Therefore, before searching for the disease-causing mutations, the coverage of the targeted sequence should be checked. Missing coverage may vary case by case, depending on the gene. In this study, we showed the *RHO* gene, when examined with CNGS, was well covered in our data set (Table S2).

More than 280 genes are linked with RP, including 25 genes are known to cause adRP^3^,^5^. Over 1000 mutations have been reported in these 25 genes^5^. Theoretically, each one accounts for a small percentage of all disease-causing genes. While, *RHO* gene has a pretty high frequency of 7.7–25% in adRP^6^,^15^,^16^; therefore, it could be treated as a “major” gene for adRP. In terms of diagnosis of the autosomal-dominant inherited disease, this study highlights the “major” gene should be prioritized for Sanger sequencing before NGS if its size is small because it has a relatively good chance of being easily identified. The human *RHO* gene has only five exons, and four pairs of primers could be used for amplifying the whole coding sequence and flanking intron sequences (Table S3). Four sequencing reactions could fully cover all the coding and flanking intron sequences, thus being a cost-effective approach for adRP diagnosis.

To our knowledge, this is the first case to report that a disease-causing mutation for autosomally dominant inherited disease could also have a reference SNP ID. In addition, we suggest the screening strategy for autosomally dominant inherited disease should be to screen the major genes for mutations before performing NGS. Thus, the findings of this study are applicable to screening mutations for RP, paving the way for gene therapy and prenatal *RHO*-causing RP diagnosis.

## Methods

### Patient Recruitment

This study conformed to the tenets of the Declaration of Helsinki. It was approved by the Ethics Committee of our hospital. Written informed consent was obtained from the recruited individuals. The study included 16 participants, including seven affected individuals, seven unaffected individuals, and two spouses (Fig. 1). All experiments were performed in accordance with the approved guidelines. Optical coherence tomography (OCT), ERG and fundus examinations were performed as routine retinal ophthalmic examinations. A five ml venous blood sample was drawn into an ethylenediaminetetraacetic acid (EDTA) sample tube. Genomic DNA was extracted from peripheral blood leukocytes using the standard phenol/chloroform extraction protocols.

### Capturing Next-Generation Sequencing and Bioinformatics Analysis

Capturing next-generation sequencing was performed as previously described^7^,^17^. The enriched libraries were sequenced on an Illumina Solexa HiSeq 2000 sequencer for paired-end reads of 100 bp. Briefly, we used the Solexa QA, the cutadapt (http://code.google.com/p/cutadapt/), SOAP aligner^32^, BWA (bio-bwa.sourceforge.net/)^33^, and GATK programs (https://www.broadinstitute.org/gatk/)^34^, to retrieve, align, and identify SNPs and insertions or deletions (InDels). The GRCh37/hg19 version of the human genome was used for sequence alignment. SNPs and InDels were annotated using the exome-assistant program (http://122.228.158.106/exomeassistant). Nonsynonymous variants were evaluated as previously described^7^,^17^. Specifically, nonsynonymous variants were evaluated through three algorithms—SIFT (http://sift.jcvi.org/), PolyPhen (http://genetics.bwh.harvard.edu/pph2/), and PANTHER (http://www.pantherdb.org/tools/csnpScoreForm.jsp)—to determine pathogenicity. Finally, from the Chinese population, 1402 individuals without obvious eye diseases were used as normal controls.

### Cosegregation study

Primers (RHO E2F/2R, Table S3) were used for amplifying the fragment harboring the mutation. PCR was performed with the genomic DNA from the family, and its product was purified using standard protocols. Sanger sequencing and RFLP were performed as standard protocols. Specifically, concerning RFLP, the purified PCR products were digested with *Bsrb* 1, which indicated the unaffected individual with 241 and 169 bp fragments, as compared to the affected individual with 241,169 and 410 bp.

### Nomenclature of mutations

For mutations, nucleotide numbering reflects cDNA numbering, with +1 corresponding to the A of the ATG translation initiation codon in the reference sequence, according to journal guidelines (www.hgvs.org/mutnomen). The initiation codon is codon 1.

### Multiple-sequence alignment and mutation analysis

Using the NCBI and UCSC websites, we obtained multiple-sequence alignment of RHO protein in various species with DNAMAN biosoftware (Lynnon Biosoft, Quebec, Canada), including Homo sapiens, Bos taurus, Rattus norvegicus, Mus musculus, and Danio rerio.

## Additional Information

**How to cite this article**: Yu, X. *et al.* Identification of a rhodopsin gene mutation in a large family with autosomal dominant retinitis pigmentosa. *Sci. Rep.* **6**, 19759; doi: 10.1038/srep19759 (2016).

## Supplementary Material

Supplementary Information

## Figures and Tables

**Table 1:** Clinical summaries of affected individuals in this study.

Individual ID	Sex	Age (Y)	Age at onset (Y)	Best corrected visual acuity		Optometry	
				OD	OS	OD	OS
IV:15	M	35	2	0.3	0.3	−6.50/−1.50 × 160°	−8.75/−2.00 × 5°
V:2	M	13	2	0.5	0.5	−5.50	−3.0
V:3	F	8	2	0.5	0.4	−1.50 × 110°	+0.75 × 140°

M, Male; F, Female; Y, Years; OD, Oculus Dexter; OS, Oculus Sinister.
