# Supplementary material for: Identification of a rhodopsin gene mutation in a large family with autosomal dominant retinitis pigmentosa
Source: Sci Rep. 2016 Jan 22;6:19759. doi: 10.1038/srep19759 (PMC4726306; doi:10.1038/srep19759)
Supplement: Supplementary Information [file srep19759-s1.pdf]

Identification of a rhodopsin gene mutation in a large family with autosomal dominant retinitis pigmentosa

Xinping Yu[1&], Wei Shi[2&], Lulu Cheng[1], Yanfang Wang[3], Ding Chen[1], Xuting Hu[1], Jinling Xu[1], Liming Xu[4], Yaming Wu[5], Jia Qu[1] and Feng Gu[1]*

[1]School of Ophthalmology and Optometry, Eye Hospital, Wenzhou Medical University, State Key Laboratory Cultivation Base and Key Laboratory of Vision Science, Ministry of Health and Zhejiang Provincial Key Laboratory of Ophthalmology and Optometry, Wenzhou, Zhejiang 325027 China

[2]Department of Ophthalmology, Beijing Children's Hospital, Capital Medical University, Beijing 100045 China

[3]Zhejiang Key Laboratory of Medical Genetics, School of Laboratory Medicine and Life Sciences, Wenzhou Medical University, Wenzhou, Zhejiang 325035 China

[4]Department of Ophthalmology, The First Affiliated Hospital of Zhengzhou, Zhengzhou University, Henan 450052 China

[5]Department of Ophthalmology, the First Affiliated Hospital of Wenzhou Medical University, Wenzhou, Zhejiang 325000 China

***Correspondence to:** Feng Gu, Eye Hospital, Wenzhou Medical University, China; 270 Xueyuan West Road, Wenzhou, Zhejiang 325027 China Phone(Fax): +86-577-8839 5300; E-mail: gufenguw@gmail.com

[&]These two authors contributed equally to this paper

Supplemental information

Table of Contents

Supplementary Figures
Figure S1

Supplementary Tables
Table S1
Table S2
Table S3
Table S4

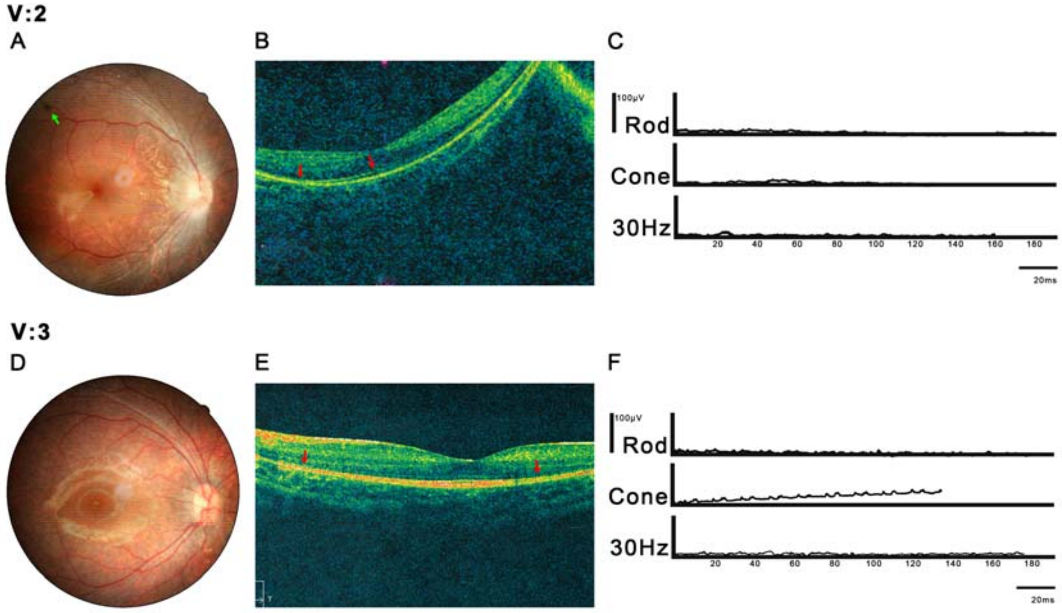

Figure S1. Clinical manifestations of RP patients

A&D. Color fundus photographs demonstrated slight intraretinal bone spicule pigmentation has been observed. B&E. OCT of the patient showed the thickness of thinning of the outer retinal layer. C&F. Non-recordable ERG has been detected except cone function of V:3 .

Table S1. Novel DNA variants

Gene name	Transcript ID	Exon	Change at c.DNA level	Change at protein level
ALMS1	CCDS42697.1	8	C.1574T>C	p.L525P
ALMS1	CCDS42697.1	8	C.1576G>C	p.E526Q
CDH15	CCDS10976.1	7	C.928A>T	p.T310S
GNAT1	CCDS2812.1	4	C.413G>A	p.R138H
GUCY2D	CCDS11127.1	2	C.890A>G	p.N297S
GUCY2D	CCDS11127.1	10	C.2165G>T	p.R722L
RP1L1	CCDS43708.1	1	C.3932A>C	p.E1311A
SNRNP200	CCDS2020.1	17	C.13859C>T	p.R287W
SPATA7	CCDS32132.1	4	C.148G>C	p.D50Y

Table S2. CNGS of *RHO* gene

Gene	Exon	Average depth	Position
RHO	1	281.99	chr3:129247576-129247937
RHO	2	317.16	chr3:129249718-129249887
RHO	3	153.01	chr3:129251093-129251259
RHO	4	160.00	chr3:129251375-129251615
RHO	5	217.83	chr3:129252441-129252570

Table S3. PCR information for *RHO*

Oligo Name	Sequence	Size (bp)	Anneal Tem.	Genome location
RHO E1F	CCAATTAGGCCCTCAGTTTCT	698	59	Chr3:129247351-129248048
RHO E1R	AGGACAGGAGAAGGGAGAAG			
RHO E2F	CCCAAGGCCTCCTCAAATC	410	61	Chr3:129249591-129250000
RHO E2R	CAGGAGACATACAAGGTCAGTG			
RHO E3-4F	TTTCCAGGGAGGGAATGTG	703	60	Chr3:129250987-129251689
RHO E3-4R	TGGGAAGTAGCTTGTCCTTG			
RHO E5F	CTCAGTCCCTGGCATCTCTA	426	62	Chr3:129252255-129252680
RHO E5R	GAGCCTATGTGACTTCGTTCAT			

Table S4. Functional effect predication for p.R135W

Protein Change	SIFT	PolyPhen2_HDIV	PolyPhen2_HVAR	LRT
p.R135W	Damaging	Probably_damaging	Probably_damaging	Deleterious
